# Supplementary material for: Atomic structures of a bacteriocin targeting Gram-positive bacteria
Source: Res Sq. 2024 Mar 27:rs.3.rs-4007122. Preprint. [Version 1] doi: 10.21203/rs.3.rs-4007122/v1 (PMC10996793; doi:10.21203/rs.3.rs-4007122/v1)
Supplement: Supplement 1 [file NIHPPrs4007122v1-supplement-1.pdf]

Supplementary Table. 1 | CryoEM data collection, refinement and validation statistics

	Pre-contraction Trunk (EMD-42959) (PDB 8V3X)	Pre-contraction Collar (EMD-42953) (PDB 8V3T)	Pre-contraction Baseplate (C6 sym.) (EMD-42957)	Pre-contraction Baseplate (C3 sym.) (EMD-42958)	Pre-contraction Baseplate (C3 sym. triplex) (EMD-42956) (PDB 8V3W)
**Data collection and processing**					
Magnification	81,000	81,000	81,000	81,000	81,000
Voltage (kV)	300	300	300	300	300
Electron exposure (e–/Å^2)	50	50	50	50	50
Defocus range (μm)	−1.0 – −4.0	−1.0 – −4.0	−1.0 – −4.0	−1.0 – −4.0	−1.0 – −4.0
Pixel size (Å)	0.55*	1.1	1.1	1.1	1.1
Symmetry imposed	C6 + helix	C6	C6	C3	C3
Initial particle images (no.)	2,345,283	202,971	414,022	414,022	414,022
Final particle images (no.)	450,732	144,368	353,620	353,620	116,539
Map resolution (Å)	2.2	2.7	2.6	2.7	2.9
FSC threshold	0.143	0.143	0.143	0.143	0.143
Map resolution range (Å)	2.1 – 2.9	2.5 – 4.0	2.3 – 5.5	2.4 – 6.0	2.5 – 6.0
**Refinement**					
Initial model used (PDB code)	*de novo*	*de novo*			*de novo*, AlphaFold2
Model resolution (Å)	2.2	2.8			3.0
FSC threshold	0.5	0.5			0.5
Map sharpening *B* factor (Å^2)	-70	-70			-70
Model composition					
Non-hydrogen atoms	75,948	76,362			116,790
Protein residues	9,696	9,720			14,709
RNA/DNA Nucleotides					
Ligands					
B factors (Å^2)					
Protein	5.62	69.73			97.53
Ligand					
R.m.s. deviations					
Bond lengths (Å)	0.004	0.008			0.004
Bond angles (°)	0.729	0.731			0.577
Validation					
MolProbity score	1.69	1.77			1.63
Clashscore	5.35	6.7			8.52
Poor rotamers (%)	3.3	4.1			2.6
Ramachandran plot					
Favored (%)	96.82	97.58			97.02
Allowed (%)	3.18	2.42			2.98
Disallowed (%)	0.00	0.00			0.00

	Post-contraction Trunk (EMD-42960) (PDB 8V3Y)	Post-contraction Collar final (EMD-42962) (PDB 8V40)	Post-contraction Collar transitional (EMD-42961) (PDB 8V3Z)	Post-contraction Baseplate final (EMD-42964) (PDB 8V43)	Post-contraction Baseplate transitional (EMD-42963) (PDB 8V41)
**Data collection and processing**					
Magnification	81,000	81,000	81,000	81,000	81,000
Voltage (kV)	300	300	300	300	300
Electron exposure (e–/Å^2)	50	50	50	50	50
Defocus range (μm)	−1.0 – −4.0	−1.0 – −4.0	−1.0 – −4.0	−1.0 – −4.0	−1.0 – −4.0
Pixel size (Å)	1.1	1.1	1.1	1.1	1.1
Symmetry imposed	C6 + helix	C6	C6	C6	C6
Initial particle images (no.)	528,409	117,846	117,846	30,712	30,712
Final particle images (no.)	65,376	12,152	12,446	11,219	11,345
Map resolution (Å)	3.6	3.9	3.6	6.1	5.6
FSC threshold	0.143	0.143	0.143	0.143	0.143
Map resolution range (Å)	3.4 – 5.0	3.4 – 6.7	3.2 – 6.6	4.8 – 9.0	4.5 – 8.5
**Refinement**					
Initial model used (PDB code)	*de novo*	*de novo*	*de novo*	*de novo*	*de novo*
Model resolution (Å)	3.7	3.9	3.5	7.9	7.6
FSC threshold	0.5	0.5	0.5	0.5	0.5
Map sharpening *B* factor (Å^2)	-70	-70	-70	-70	-70
Model composition					
Non-hydrogen atoms	82,290	76,422	76,422	95,334	95,286
Protein residues	10,590	9,732	9,732	12,066	12,060
RNA/DNA Nucleotides					
Ligands					
B factors (Å^2)					
Protein	118.87	102.22	66.44	364.20	331.20
Ligand					
R.m.s. deviations					
Bond lengths (Å)	0.004	0.003	0.003	0.002	0.002
Bond angles (°)	0.748	0.521	0.505	0.473	0.521
Validation					
MolProbity score	1.94	1.61	1.51	1.80	1.99
Clashscore	14.29	8.29	7.08	13.70	18.82
Poor rotamers (%)	3.8	0.2	2.3	0.1	0.3
Ramachandran plot					
Favored (%)	95.98	97.10	97.41	97.15	96.64
Allowed (%)	4.02	2.90	2.59	2.85	3.36
Disallowed (%)	0.00	0.00	0.00	0.00	0.00

* Particles used for the reconstruction were extracted from un-binned micrographs collected in super-resolution mode (pixel size of 0.55 Å).

Reference:

1 Taylor, N. M. *et al.* Structure of the T4 baseplate and its function in triggering sheath contraction. *Nature* **533**, 346-352 (2016). https://doi.org:10.1038/nature17971

2 Powell, A. J., Liu, Z. J., Nicholas, R. A. & Davies, C. Crystal structures of the lytic transglycosylase MltA from N.gonorrhoeae and E.coli: insights into interdomain movements and substrate binding. *J Mol Biol* **359**, 122-136 (2006). https://doi.org:10.1016/j.jmb.2006.03.023

3 Sekiya, H., Tamai, E., Kawasaki, J., Murakami, K. & Kamitori, S. Structural and biochemical characterizations of the novel autolysin Acd24020 from Clostridioides difficile and its full-function catalytic domain as a lytic enzyme. *Mol Microbiol* **115**, 684-698 (2021). https://doi.org:10.1111/mmi.14636

4 Kim, B. *et al.* Enterococcus faecium secreted antigen A generates muropeptides to enhance host immunity and limit bacterial pathogenesis. *Elife* **8** (2019). https://doi.org:10.7554/eLife.45343

5 Ge, P. *et al.* Action of a minimal contractile bactericidal nanomachine. *Nature* **580**, 658-662 (2020). https://doi.org:10.1038/s41586-020-2186-z

6 Wang, J. *et al.* Cryo-EM structure of the extended type VI secretion system sheath-tube complex. *Nat Microbiol* **2**, 1507-1512 (2017). https://doi.org:10.1038/s41564-017-0020-7

7 Fokine, A. *et al.* The molecular architecture of the bacteriophage T4 neck. *J Mol Biol* **425**, 1731-1744 (2013). https://doi.org:10.1016/j.jmb.2013.02.012

8 Jiang, F. *et al.* Cryo-EM Structure and Assembly of an Extracellular Contractile Injection System. *Cell* **177**, 370-383 e315 (2019). https://doi.org:10.1016/j.cell.2019.02.020

9 Kizziah, J. L., Manning, K. A., Dearborn, A. D. & Dokland, T. Structure of the host cell recognition and penetration machinery of a Staphylococcus aureus bacteriophage. *PLoS Pathog* **16**, e1008314 (2020). https://doi.org:10.1371/journal.ppat.1008314

10 Jumper, J. *et al.* Highly accurate protein structure prediction with AlphaFold. *Nature* **596**, 583-589 (2021). https://doi.org:10.1038/s41586-021-03819-2
